# The Hunger Strike—A Psychological Problem

**Published:** 1912-08-10

**Authors:** 


### The Hospital

The Workers' Newspaper of Administrative Medicine and Institutional Life, Administration, National Insurance and Health.

No. 1362, Vol. LII. SATURDAY, AUGUST 10, 1912. PRICE ONE PENNY.

### THE HUNGER STRIKE—A PSYCHOLOGICAL PROBLEM.

It is difficult, if not impossible, for men to approach the consideration of this question with a perfectly detached and unbiassed mind. One reason for this is obvious, and is, in itself, a proof that the age of chivalry is not yet past. The sex factor is powerful, and it is impossible to evade the influence of the unconscious bias which it gives to the masculine mind. Women are not, in this instance, affected by it, and the strongest and most contemptuous expressions of opposition to the strike fall from feminine lips. The educated man revolts from the idea of inflicting mental pain and physical suffering upon delicate women who are his equals in education and refinement. To men of sensitive and artistic temperament it is horrible to think of women whom they know and respect being subjected to forcible feeding. They have no practical knowledge of what that really means, and their imagination invests that operation, unpleasant as it undoubtedly is, with terrors which are non-existent. They cannot consider the question apart from the individuals who have made its consideration necessary. Would they be so concerned if it had been male prisoners who were attempting to carry out this method of evading the law?

Then, again, those concerned with the administration of justice are liable to have their judgment warped by professional bias. Those of us who are subject to this are perhaps unaware how great is its power. It overwhelms the unconscious bias of sex. Those connected with prison administration perceive that this method of opposition to the law is capable of a wide extension. For the vast majority of those under their charge who might employ it they feel no sympathy whatever. The bent of the official mind is not towards leniency in dealing with such innovations, however introduced. Even when uninfluenced by this professional bias there are few of us who would not be equally unsympathetic if the "hunger strike" were adopted by the ordinary inmates of our prisons. Even those who advocate that the women of late undergoing forcible feeding should be set at liberty would hesitate before admitting that the same treatment should be applied to all prisoners who try to starve themselves in order to call attention to what they believe to be a vital principle. This is only evidence of our failure to dissociate the nature of an act from the personality of the actor. In each case it is an attempt to escape from the legal consequences of an infringement of the law. The cases, however, are not viewed from the same standpoint. The wisdom of many, crystallised into words by the wit of one, expresses how universal is this inability to do even-handed justice between different individuals: one man may steal a horse, but another must not look over the hedge.

It is evident from the expressions of opinion which we meet in print that many of those who are in sympathy with the movement for which the hunger-strikers are suffering are equally unfitted to apply the first principles of scientific investigation to the problem. Doubts have even been expressed as to whether it is right to hinder a mental patient from starving himself. What particular sanctity is attached to this form of suicide? We read harrowing accounts of the dangers and horrors of forcible feeding. How dreadful a tale could be told of the giving of medicine by a mother to an unwilling child, if described by a writer with a glowing vocabulary, a keen prejudice against the mother, and an ardent sympathy with the child in its refusal to take physic! What a cruel picture might be drawn of the washing of the face of an unruly urchin! Much that is said with regard to this part of the question indicates the undue prominence of feeling uncontrolled by intellect. That way lies hysteria.

We admire the high character and devotion of the hunger-strikers. We give them full credit for their conviction that they are suffering to advance a great cause. To the psychologist every phase of such a movement has an interest. He recognises that the ladies are satisfied that the principle in question justifies their attempting voluntary starvation. He is conscious how difficult it is to distinguish between the false and the true. He endeavours to look upon their action from an unpersonal standpoint, though aware that he too may not be free from the unconscious bias. His life work teaches him that such convictions are not incompatible with and may even be dependent upon weak reasoning power. He sees daily that a willingness and even an anxiety to undergo various forms of martyrdom can be inspired and sustained by a false belief arising from diseased mental action. Refusal of food is a common symptom in the mental hospital.

It may be due simply to a desire to terminate existence. It may arise from delusions with regard to the food or those who offer it. It may be dependent upon delusions with respect to the ability of the body to deal with it. It may be kept up by the belief that others will be injured if nourishment be allowed to pass the patient's lips. He may hear a voice telling him that the Almighty has forbidden him to eat. The motive may be one which, while it cannot be recognised as sufficient excuse for suicide, yet merits our respect. There are other reasons which do not so appeal to us. A patient of an hysterical temperament may refuse food and require forcible feeding because she desires to share in the attention and notice which is paid to another who is so fed. It is not by any means unknown for a patient to refuse food under the idea that discharge is thus rendered necessary.

